# Links between Vitamin K, Ferroptosis and SARS-CoV-2 Infection

**DOI:** 10.3390/antiox12030733

**Published:** 2023-03-16

**Authors:** Jarosław Nuszkiewicz, Paweł Sutkowy, Marcin Wróblewski, Marta Pawłowska, Roland Wesołowski, Joanna Wróblewska, Alina Woźniak

**Affiliations:** Department of Medical Biology and Biochemistry, Faculty of Medicine, Ludwik Rydygier Collegium Medicum in Bydgoszcz, Nicolaus Copernicus University in Toruń, 24 Karłowicza St., 85-092 Bydgoszcz, Poland

**Keywords:** antioxidants, COVID-19, ferroptosis, oxidative stress, redox homeostasis, SARS-CoV-2, vitamin K

## Abstract

Ferroptosis is a recently discovered form of programmed cell death. It is characterized by the accumulation of iron and lipid hydroperoxides in cells. Vitamin K is known to have antioxidant properties and plays a role in reducing oxidative stress, particularly in lipid cell membranes. Vitamin K reduces the level of reactive oxygen species by modulating the expression of antioxidant enzymes. Additionally, vitamin K decreases inflammation and potentially prevents ferroptosis. Severe acute respiratory syndrome coronavirus 2 (SARS-CoV-2) infection leading to coronavirus disease 2019 (COVID-19) is associated with oxidant–antioxidant imbalance. Studies have shown that intensified ferroptosis occurs in various tissues and cells affected by COVID-19. Vitamin K supplementation during SARS-CoV-2 infection may have a positive effect on reducing the severity of the disease. Preliminary research suggests that vitamin K may reduce lipid peroxidation and inhibit ferroptosis, potentially contributing to its therapeutic effects in COVID-19 patients. The links between ferroptosis, vitamin K, and SARS-CoV-2 infection require further investigation, particularly in the context of developing potential treatment strategies for COVID-19.

## 1. Introduction

Coronavirus disease 2019 (COVID-19), caused by the severe acute respiratory syndrome coronavirus 2 (SARS-CoV-2), continues to pose a significant challenge both medically and socially [1]. The first cases of SARS-CoV-2 infection were observed at the end of 2019 in Wuhan, the capital city of Hubei Province, China [2]. The infection spread rapidly and eventually led to a pandemic declared by the World Health Organization (WHO) on 11 March 2020 [1]. The symptoms of COVID-19 may vary significantly, and the course of the disease can range from mild to severe. The most common symptoms include fever, cough, acute lung injury, and septic shock, and even acute respiratory distress syndrome (ARDS) [3,4]. However, many other symptoms, such as loss of taste or smell, sore throat, congestion or runny nose, headache, muscle or body aches, nausea or vomiting, and diarrhea, have been reported in individuals with COVID-19 [1,2]. Infection caused by SARS-CoV-2 has been associated with an increased risk of coagulopathies [5]. Hemostasis disorders may lead to disseminated intravascular coagulopathy (DIC) and severe stroke bleeding [5].

One of the factors regulating the functioning of the coagulation system is vitamin K [6]. The result of vitamin K deficiency may be easy bruising and spontaneous bleeding in the case of significant deficiencies [7]. So far, vitamin K has been associated mainly with coagulation process regulation and the skeletal system’s homeostasis. The latest scientific reports indicate that vitamin K has antioxidant properties [8]. Many pathological conditions, including infectious diseases, lead to redox imbalance. Increased reactive oxygen species (ROS) generation is also observed during COVID-19 [9]. Oxidative stress may be related to both the severity of the disease and its symptoms as well as prognosis and post-COVID-19 syndrome [10].

The result of increased oxidative stress during SARS-CoV-2 infection may be ferroptosis [11]. Ferroptosis is a form of programmed cell death associated with the accumulation of iron and lipid hydroperoxides (LOOHs) [11]. Ferroptosis may be a form of cell response and an attempt to defend against SARS-CoV-2 infection, but it also contributes to the faster spread of the virus [12]. One of the therapeutic strategies to reduce ferroptosis in the course of COVID-19 may be supplementing with vitamin K due to its antioxidant properties. Vitamin K, as a lipophilic molecule, can reduce the formation of LOOHs.

This article aims to indicate the relationship between increased oxidative stress in the course of COVID-19, ferroptosis, and the potential therapeutic use of vitamin K to reduce the negative impact of ROS on cells. This publication is an analysis of the latest scientific literature from the PubMed, Google Scholar, and Web of Science databases.

## 2. Oxidant–Antioxidant Balance and Lipid Peroxidation

The consequence of oxygen consumption in order to live is the generation of ROS in cells. The term ROS encompasses both free oxygen radicals and nonradical species. Major ROS include, among others, superoxide anion radical (O_2_^•−^), hydrogen peroxide (H_2_O_2_), hydroxyl radical (HO^•^), and nitric oxide (^•^NO), which is also a reactive nitrogen species (RNS) [13]. ROS are generated during normal functioning of cells [14]. Their main cellular sources are mitochondria and NADPH oxidases (NOXs) [15,16]. If the equilibrium between ROS generation and antioxidant defense mechanisms is distorted in favor of oxidants, this condition leads to what is known as oxidative stress [17,18], the potential consequence of which may be cell damage at the molecular level [19], including oxidation of proteins, DNA, and lipids [20]. Oxidative stress has been proven to play an essential role in the pathogenesis of a number of diseases [21,22]. However, ROS also have a role in regulating signaling pathways, affecting the course of biological processes [20,23]. According to the latest update of the concept of oxidative stress, a distinction is made between oxidative distress when elevated generation of ROS leads to molecular damage and oxidative eustress when ROS occur at physiological levels, playing an important role in redox signaling [24]. The key factors in redox signaling are considered to be O_2_^•−^ and H_2_O_2_. Their generation occurs under the control of growth factors and cytokines by more than 40 enzymes [24].

One of the processes responsible for oxidative stress is lipid peroxidation (LPO) [25]. In the course of this process we distinguish three stages: initiation, propagation, and termination [26,27]. The cycle begins with detachment of a hydrogen atom from a methylene group molecule of polyunsaturated fatty acids (PUFAs) and formation of a lipid free radical (L^•^). Then, rearrangement occurs of double bonds and the formation of a more stable radical containing conjugated dienes (CD) [28,29,30]. The initiating factor for LPO may be, among others, HO^•^ and a hydroperoxyl radical (HO_2_^•^) [26,31]. During the propagation stage, the lipid radical reacts with oxygen, forming a peroxyl radical (LOO^•^). It is a radical of high energy that can detach a hydrogen atom from a lipid molecule and transform into a LOOH molecule. This reaction is accompanied by the formation of another L^•^. LOOHs, formed during the propagation stage, are the main primary products of the LPO process [26]. They can then undergo one-electron or two-electron reduction. During iron-mediated one-electron reduction and oxygenation (in accordance with the equation: LOOH + Fe^2+^ → O_2_ → OLOO^•^ + OH + Fe^3+^), epoxyallylic peroxyl radicals (OLOO^•^) are formed, causing further free radical-mediated chain peroxidation [32]. At the termination stage, the free radicals (L^•^, LOO^•^) formed react with one another, creating products which are not free radicals [28]. Final transformations of LPO products, during β-elimination and decomposition of PUFAs derivatives, lead to the formation of secondary products of the peroxidation process, among others, malondialdehyde (MDA), 4-hydroxynonenal (4-HNE) and isoprostanes [29,33]. LPO products, including LOOHs and aldehydes, are highly reactive, may cause damage of proteins and DNA, and cause selective alterations in cell signaling [34].

Oxidation of PUFAs can also occur with the participation of enzymes, among others, lipoxygenases (LOXs) and cyclooxygenases (COXs) [35]. It has been demonstrated that the production of LOOHs in the membranes of neoplastic cells, with the participation of LOX, enhances ferroptosis induced by erastin (an inhibitor of the cystine/glutamate antiporter) and RSL3 (an inhibitor of glutathione peroxidase 4 (GPx4)) [36].

The antioxidant defense system present in cells, which is responsible for removing ROS, is formed by enzymes and non-enzymatic antioxidants. The enzyme barrier includes, among others, superoxide dismutase (SOD), GPx, catalase (CAT), and thioredoxin (Trx). Non-enzymatic ROS scavengers include vitamins or their analogs (among others, vitamins C, A, E; flavonoids, coenzyme Q10 (CoQ10)), metabolites (e.g., melatonin, bilirubin), and minerals (e.g., zinc, selenium) [37]. An antioxidant function in membranes is also played by vitamin K [38]. The Trx system and glutathione antioxidant system are the two major thiol-dependent antioxidant systems in mammalian bodies [39]. The Trx system, including Trx, thioredoxin reductase (TrxR), thioredoxin peroxidase (TPx), and NADPH, participates in the regulation of gene expression and modulation of cell signaling pathways, but also in the detoxification of LOOHs [40]. SOD neutralizes O_2_^•−^ by its dismutation to hydrogen peroxide and oxygen. In mammals, three SOD isoforms have been identified: CuZn isoform (CuZn-SOD or SOD1) present in the cytosol and nucleus; manganese SOD (Mn-SOD or SOD2) localized in mitochondria; and extracellular CuZn-SOD (EC-SOD or SOD3) [41,42]. CAT is mainly present in peroxisomes [14]. It contains a molecule of ferric ion at its active site and conducts dismutation of H_2_O_2_ into oxygen and water. Besides this basic function, CAT also participates in the decomposition of, among others, hydroperoxides, methanol, ethanol, azide, and formate [43,44]. Some studies confirm that CAT may decompose peroxynitrite [45] and oxidize nitric oxide to nitrite [43,44].

GPx is also involved in the removal of H_2_O_2_ and LOOHs [46,47], in accordance with the following reactions [48]:

H_2_O_2_ + 2GSH → 2H_2_O + GSSG (1), and LOOH + 2GSH → LOH + GSSG + H_2_O (2) (GSH—reduced glutathione, GSSG—oxidized glutathione).

To date, eight different GPxs have been detected in humans. In five of them (GPx1-4 and GPx6), a selenocysteine residue occurs at the active site [49]. Enzymes of the GPx family, especially GPx4, are important regulators of the of LOOHs in cells. They catalyze the two-electron reduction of LOOH leading to the formation of redox-inert alcohol (2) [50]. The co-substrate used during LOOH reduction to alcohol is GSH [51]. GPx4 activity is detected in the cytosol, cell nucleus, and mitochondria. This enzyme is considered as a key regulator of ferroptosis. GPx4 can reduce peroxidized complex lipids and it also silences LOX [49]. By detoxifying hydroperoxides in membrane lipids, GPx4 reduces the degree of damage to membrane function and prevents the generation of reactive products of the peroxidation process [52].

## 3. Ferroptosis Mechanisms and Health Implications

**Figure 1:**
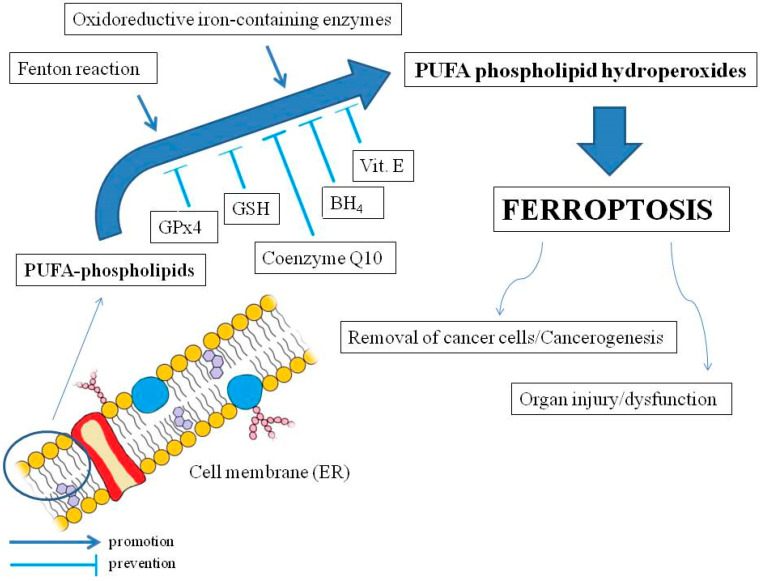
The main mechanisms of ferroptosis and the effects on the human organism. Polyunsaturated fatty acids (PUFAs) in membrane phospholipids (mainly endoplasmic reticulum, ER) are oxidized by reactive oxygen species from the Fenton reaction or as a result of activity of oxidoreductive iron-containing enzymes. The oxidation may lead to ferroptosis by accumulation of PUFA phospholipid hydroperoxides, which may be inhibited by soluble glutathione peroxidase 4 (GPx4) and glutathione (GSH), as well as lipophilic membrane protectors such as coenzyme Q10, tetrahydrobiopterin (BH4), and vitamin E. Ferroptosis may be implicated in cancer cell death but may also promote carcinogenesis as well as organ injuries and dysfunctions (slightly modified cell membrane diagram, courtesy of Holly Barker/The Science Hive).

In 2012, Brent Stockwell with his colleagues, including Scott Dixon, proposed the concept of the iron-dependent form of regulated cell death, which is oxidative-related, termed ferroptosis, which is distinct from apoptosis, unregulated necrosis, and necroptosis (regulated necrosis). The main morphological features of ferroptosis are mitochondrial shrinkage accompanied by increased mitochondrial membrane density and a degenerated mitochondrial inner membrane (i.e., electron transport chain) without changes in the nucleus [53]. The condition for ferroptosis is the oxidation of membrane lipids (particularly endoplasmic reticulum, ER) and the disruption of mechanisms that prevent the accumulation of oxidized lipids. Therefore, ferroptosis should not be considered a type of oxidative stress but rather an accumulation of lethal LOOHs located in cell membranes [54] (see Figure 1).

Exceptionally implicated in ferroptosis are phospholipids (PLs) characterized by a high content of PUFAs. Formation of PUFA hydroperoxides in complexes with phospholipids (PLs–OOH) promotes ferroptosis [54]. However, not all PLs contribute equally to ferroptosis. Phosphatidylethanolamines with one fatty acyl tail derived from arachidonic acid or adrenic acid (docosatetraenoic acid) are more closely related to ferroptosis than other PLs [55]. Occasionally, PLs with two PUFA tails are also observed. They are most susceptible to ferroptosis [56]. It was also found that free PUFAs probably do not affect ferroptosis [57], similarly to PUFA hydroperoxides, for example, after their cleavage from PLs by Ca^2+^-independent phospholipase A2β (iPLA2β) [58]. In turn, the incorporation of monounsaturated fatty acids (MUFAs) into PLs entails an anti-ferroptotic effect [59], and saturated fatty acids in complex with PLs have no impact on this phenomenon [54].

Oxidation of PLs in ferroptosis is associated with the transition of iron ions in the oxidation state II (Fe^2+^), which results in a Fenton reaction and ROS production, as well as with iron-containing enzymes, such as LOXs [60] (they generally require Fe^3+^ for activity [61]) or, e.g., cytochrome P450 oxidoreductases [62] (see Figure 1). As for cellular organelles, mitochondria may initiate or amplify ferroptosis induced by cysteine deficiency associated with GSH depletion. Additionally, those organelles play a central role in oxidative metabolism. Electron leakage from the mitochondrial electron transport chain is manifested by the formation of O_2_^•−^ and H_2_O_2_, which can then react with Fe^2+^ to drive Fenton chemistry and LPO [54]. Meanwhile, as already mentioned, the relationship between ferroptosis and significant morphological changes of mitochondrial crista is a fact [53]. Moreover, inhibiting the mitochondrial electron transport chain also attenuates ferroptosis induced by cysteine starvation, as does depletion of mitochondria [54]. LPO, beyond its implication in ferroptosis, can be a link with other forms of regulated cell death, especially apoptosis and autophagy-dependent death, but also: pyroptosis, necroptosis, parthanatos, and netotic cell death [63]. Iron abundance in the cell depends, in turn, on its transport with blood from the gut. This mainly involves transferrin (TF) and its cell receptors [64]. Cellular iron availability, in turn, is regulated by ferritin (FER), which stores it in the form of Fe^3+^ and prevents ferroptosis [65]. Some proteins can act on ferroptosis by affecting FER concentration. For example, kinase ataxia telangiectasia inhibits FER synthesis and thus may promote ferroptosis [66]. Other mechanisms for regulating cellular iron pool and ferroptosis are related to iron export (ferroportin (FPN) and exosomes) [67].

Fe^2+^ availability in the cell is also associated with low-molecular-weight compounds, including GSH. Iron storage in FER requires the formation of a GSH–iron complex [68]. Thus, depletion of GSH can promote ferroptosis not only by facilitation of ROS production in the Fenton reaction but also by inhibition of GPx4, which is particularly involved in the prevention of LPO. GSH and GPx4 are considered major inhibitors of ferroptosis [69]. Interestingly, GPx4 degradation, e.g., through protein ferroptosis-inducer-56 (FIN56) [70] or chaperone-mediated autophagy [71], is more conducive to ferroptosis than inhibition of the enzyme. Deficiency of GSH and thus promotion of ferroptosis may also result from inhibition of GSH production, e.g., due to inhibition of glutamate–cysteine ligase [72] or blocking of cystine uptake in membrane system Xc^−^, a cystine/glutamate antiporter system, but also as a result of GSH efflux [69] or increased catabolism of cysteine (dioxygenase 1, CDO1) [73]. Moreover, it was reported that the mentioned ligase increased cell resistance to ferroptosis not only by contribution to GSH synthesis but also as a result of glutamate conversion to γ-glutamyl peptides, which suggests that glutamate can promote ferroptosis [72].

Important endogenous suppressors of ferroptosis are also lipophilic antioxidants, especially CoQ10. It is present in different cell membranes, including mitochondria, and strongly protects against LPO. After lipoperoxidation, it can be recovered with NADPH [74]. Suppressing LPO and, thus, ferroptosis is also associated with another endogenous lipophilic antioxidant, tetrahydrobiopterin (BH_4_) [56]. Exogenous antioxidants, e.g., dietary ingredients such as vitamin E, may also protect against ferroptosis [75]. Moreover, ferroptosis may be inhibited by dihydroorotate dehydrogenase (DHODH), reducer of CoQ10 in mitochondria [76], and interleukin-4-induced-1 (IL4I1) via indole-3-pyruvate (In3Py) synthesis [77].

Ferroptosis is implicated in many phenomena in the human organism (see Figure 1). For instance, attenuation of ferroptosis in CD4^+^ T cells with a selenium-rich diet (selenocysteine occurs in the active site of GPx4) triggered an increase in the number of memory B cells and long-lasting viral immunity [78]. An in vitro study demonstrated that excessive uptake of PUFAs by tumor cells in an acidic environment can lead to their ferroptosis, while a diet rich in PUFAs promoted ferroptosis of cancer cells in mice [79]. In turn, CD8^+^ T cells kill tumor cells in the way of ferroptosis by releasing interferon gamma (IFN-γ) and arachidonic acid, which results in increased incorporation of PUFAs into PLs [80], whereas a cholesterol-rich diet aided in increased PUFAs uptake by CD8^+^ T cells and their death by ferroptosis, which may promote cancer progression [81]. Moreover, ferroptosis may be implicated in multi-organ dysfunction syndrome or organ injury as a result of iron overload or other reasons as well (marked in brackets), e.g., brain (neurodegenerative diseases), heart, liver (hepatitis C infection), kidney, and lungs (*Pseudomonas aeruginosa*, SARS-CoV-2 infections). The relationship between ferroptosis and inflammation is otherwise unclear [54].

The described regulatory mechanisms of ferroptosis (LPO caused by iron-mediated oxidative stress and the GPx4-GSH inhibition pathway) may be crucial for the treatment of COVID-19 [11,12], including post COVID-19 syndrome [10], since pathological condition in SARS-CoV-2 infections is strictly associated with mitochondrial dysfunction, iron overload, oxidative stress, and inflammation [9,10,11,12].

## 4. Ferroptosis and COVID-19

COVID-19 manifests itself in many complications as well as physiological and biochemical alterations. These include, among others, increased concentrations of proinflammatory CD4^+^ T and CD8^+^ T cells, a massive release of cytokines (so-called cytokine storm), increased coagulation state, hemoglobin damage, and dysregulation of iron homeostasis, including iron overload, which is likely a significant factor in COVID-19 pathogenesis [82]. Unregulated ferroptosis may partly explain the cell degeneration and tissue damage of lung disease pathogenesis [83]. Thus, molecular interactions between systemic and cellular iron regulation and the inflammatory process is a new area of research crucial for understanding the pathogenesis of COVID-19.

**Figure 2:**
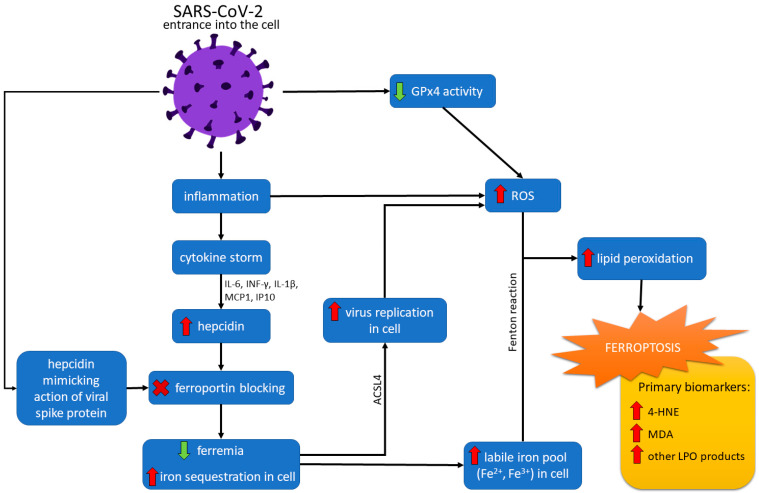
Ferroptosis pathways in SARS-CoV-2 infection. After entering the cell, the SARS-CoV-2 virus causes an inflammatory state, resulting in a cytokine storm. The effect of the cytokine storm, mediated with interleukin 6 (IL-6), interferon-gamma (IFN-γ), interleukin 1-beta (IL1-β), interferon-inducible protein 10 (IP10), and monocyte chemotactic protein 1 (MCP1) is an increased level of hepcidin. The action of hepcidin is downregulating ferroportin and determining hypoferremia and increased sequestration of iron in the cell. The virus can also imitate hepcidin action by a spike protein. Increased cellular iron level and catalytic activity of acyl-coenzyme A synthetase long-chain family member 4 (ACSL4) promotes virus replication and increases reactive oxygen species (ROS) formation. Downregulated action of GPx4 caused by the virus also leads to increased ROS production. Moreover, high cellular iron concentration can form free radicals through Fenton and Haber-Weiss reactions. ROS reacting with lipids increase lipid peroxidation (LPO), which finally causes ferroptosis.

The maintenance of iron homeostasis is crucial for the organism. Interestingly, both low and high iron levels increase the risk of infection. Elevated serum FER, low serum iron, and low transferrin levels within three days of intensive care unit admission have been observed in more than 75% of critically ill patients, indicating the importance of iron and related proteins [84]. In response to SARS-CoV-2 infection, iron metabolism dysfunction was observed in many patients. Host cells’ increased cellular metabolism and optimal iron levels are required for viral replication [85]. The hepcidin/ferroportin axis significantly regulates systematic iron [86,87]. Hepcidin, in a regular expression, has a protective function against infections. However, its overexpression in COVID-19 can be detrimental and associated with various disease symptoms and a worse prognosis [88,89]. Hepcidin essentially downregulates FPN and therefore determines hypoferremia and iron sequestration at the cellular level [86]. Due to the high concentration of iron inside the cell, free cellular iron (Fe^3+^) can easily form free radicals (e.g., through Fenton and Haber-Weiss reactions). Increased Fe^3+^ concentration is linked with FER overloading, thus leading to ferroptosis (see Figure 2) [86,90].

The SARS-CoV-2 infection causes high levels of IFN-γ, interleukin 1-beta (IL1-β), interferon-inducible protein 10 (IP10), and monocyte chemotactic protein 1 (MCP1) [87]. Therefore, stimulation of these cytokines may cause hepcidin synthesis resulting in the accumulation of iron in macrophages [87]. Moreover, the infection leads to an inflammatory state in which IL-6 stimulates the synthesis of FER and hepcidin [91,92]. The SARS-CoV-2 virus induces iron overload by promoting the overexpression of hepcidin [91,92]. The possible homology and evolutionary connections between the virus spike protein and hepcidin are the subject of research but remain unclear [92]. SARS-CoV-2 can probably imitate the action of hepcidin, increasing circulating and tissue FER. This induces serum iron deficiency and lowered hemoglobin [86,89]. It was noted that increased serum levels of hepcidin and FER are indeed associated with the severity of SARS-CoV-2 infection [92]. Otlu et al. [93] observed increased serum FER levels in critical COVID-19 patients. Excessively high ferritinemia activates mitochondrial dysfunction, leading to iron-mediated oxidative stress and aggravation of proinflammatory cytokine release [94]. Moreover, elevated FER levels trigger nuclear receptor co-activator 4 (NCOA4), mediating ferritinophagy. NCOA4 overexpression enhances FER degradation and leads to iron release. Iron converts the LOOH into hydroxyl radicals via the Fenton reaction, ultimately inducing ferroptosis and promoting cell damage [95]. Transferrin receptor-1 (TfR-1) can facilitate the entrance of iron held by transferrin into the cells [87]. TfR-1 is an ideal portal for the entry of various microorganisms into the cells [88]. It was observed that TfR-1 protein concentration in the lungs is significantly exalted in viral infection [87]. The virus can interact precisely with this receptor using its spike protein [94].

Ferroptotic cell death is a strategy for defense against virus infections [12]. However, increasing evidence shows that cell death promotes the release and spread of the virus [96]. Kung et al. [96] studied the role of acyl-coenzyme A synthetase ACSL4 in viral replication. They indicated that the catalytic activity of ACSL4 is crucial for viral replication and virus-induced ferroptosis. Wang et al. [97] observed higher expression of ACSL4 in SARS-CoV-2 infected placental tissues. Researchers suggest that the virus recruits ACSL4 for ROS formation and promotion of LPO for viral replication. Consequently, excessive LPO, GSH depletion, and a decreased level of GPx4 led to ferroptotic cell death and the release of the virus. It was also noted that ferroptosis and ACSL4 inhibitors significantly inhibit viral replication [96]. Wang et al. [97] claim that ACSL4 is a good marker of ferroptosis because it activates long-chain PUFAs for phospholipid biosynthesis preferentially and fuels the ferroptotic process. It has also been considered the primary determinant for dictating ferroptosis sensitivity and reshaping cellular lipid composition [97]. The virus suppresses mRNA expression of ferroptosis-associated GPx4, DNA synthesis-related TrxR, and endoplasmic reticulum selenoproteins [95]. Due to the lack of GPx4, GSH cannot be peroxidized to reduce the ROS of lipids formed in the Fenton reaction. Thus, ROS accumulation causes LPO and ferroptosis (see Figure 2) [90].

Inflammation, oxidative stress, and altered iron homeostasis are merged at the systemic level and play a vital role in COVID-19 pathogenesis and progression. A primary consequence of iron-dependent ferroptosis in SARS-CoV-2 infection might include cognitive impairment and loss of taste and smell. Moreover, iron-associated platelet mitochondrial dysfunction in the blood is associated with hypercoagulopathy, which is generally noticed in patients with COVID-19 [94]. SARS-CoV-2 primarily attacks the respiratory system, but the virus also causes dysfunction of other organs, such as the liver, kidneys, or heart [12]. Jacobs et al. [98] reported the accumulation of oxidized phospholipids in myocardial and kidney tissue in COVID-19 infection. This indicates that ferroptosis contributes to some forms of ischemia-reperfusion injury and is a negative factor in COVID-19 heart injury and multiple organ failure. Moreover, the brains of COVID-19 patients appear susceptible to ferroptosis [99]. Essential factors of ferroptosis are iron and unsaturated fatty acids. Iron is the most abundant trace metal in the brain. It is needed for proper cellular metabolism. The brain is sensitive to oxidative stress and LPO due to its high PUFAs levels. Neuronal cell membranes are rich in PUFAs and cholesterol and susceptible to ROS-mediated oxidation triggered by the cytokine storm in SARS-CoV-2 infection [99]. Skesters et al. [100] observed higher levels of oxidative stress indicators (MDA, 4-HNE), which are primary markers of ferroptosis [99].

## 5. Ferroptosis as a Therapeutic Target in COVID-19

The COVID-19 pandemic has become a considerable challenge and public health problem. Based on the mechanisms described above, it is reasonable to conclude that there is a link between ferroptosis and COVID-19. Since ferroptotic cell death may be involved in the pathogenesis of COVID-19, comprehending initiation of this process as well as its underlying regulatory mechanisms has potential therapeutic significance. Consequently, the need for effective therapeutic strategies against COVID-19 has become a pressing issue. Inhibiting ferroptosis may provide reliable methods for treating COVID-19 [91,101]. The crucial regulatory targets of ferroptosis include system Xc^−^ activity, the intracellular labile iron pool, GPx4 activity, GSH production, LPO, and phosphatidylethanolamine (PE) biosynthesis [99]. System Xc^−^, involved in GSH synthesis, is part of the Xc-GSH-GPx4 system responsible for eliminating LPO effects [102]. Restriction of the Xc^−^ system reduces cysteine absorption, resulting in GSH deficiency and the deposition of LOOHs. It is potential, but not yet verified, that ferroptosis could be significantly reduced by mediating GPx4 by selenium supplementation [102]. It is also suggested that ACSL4 could be a target for combating viral infections, and the use of ACSL4 inhibitors, such as rosiglitazone and pioglitazone, can decrease the viral load of coronaviruses [96].

Moreover, it has been proposed that drugs that enhance the GPx4-GSH axis and ultimately lead to iron depletion in the unstable pool may be candidates for COVID-19 treatment in response to the ferroptosis manifestation [103]. Therefore, GSH supplementation may be a beneficial adjunctive therapy in COVID-19 [85]. Moreover, iron chelators and lipophilic antioxidants may be helpful adjunctive therapies in treating COVID-19 [12,103]. Iron chelators reduce inflammation and prevent coronaviruses from binding to the receptors they use to enter host cells [103]. Edeas et al. [83] suggest that approved iron chelators, ferroptosis inhibitors, hepcidin, and erythropoietin modulators may be considered in addition to treating inflammation. Linking anti-inflammatory cytokines and interfering with iron ions metabolism to improve patients’ immunity should be explored in future studies [12]. Therapeutics of iron metabolism may provide benefit as potential drugs to inhibit viral infection exacerbation caused by cell death. Some antioxidants, like butylated hydroxytoluene or vitamin E, are recognized as both modulators of ferroptosis and COVID-19 infection-fighting support [104]. The pharmacological intervention of the ferroptosis course demonstrates promising therapeutics for virus infection prevention and control. However, the exact antiviral mechanism requires further analysis to provide essential research data. Preliminary research results suggest that therapy with vitamin K may be promising due to its antioxidant properties.

## 6. A Brief Overview of Vitamin K

**Figure 3:**
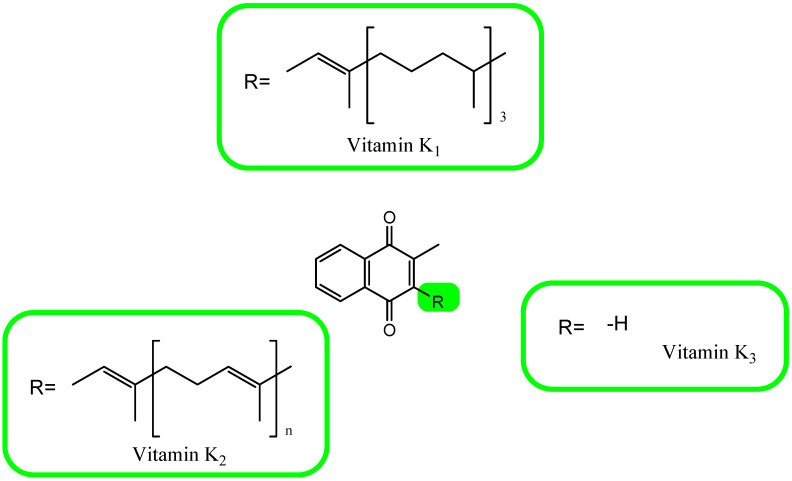
The chemical formula of vitamin K.

The concept of vitamin K, discovered in 1929, refers to compounds that are derivatives of a synthetically obtained provitamin called 2-methyl-1,4-naphthoquinone (menadione, K_3_). The main source of vitamin K for humans are plants that synthesize it in the form of phylloquinone, phytomenadione, or phytonadione, i.e., vitamin K_1_. This form of vitamin has a side chain at position 3 consisting of four isoprenyl residues, the last three of which are saturated. A chain with such a structure is called a phytyl residue [105,106,107,108,109,110]. Vitamin K_2_ is made up of a group of derivatives containing a chain of unsaturated isoprenyl residues in the position 3 naphthoquinone ring. These compounds are menaquinones (MK). Twelve different MK characters (MK-4 to MK-15) have been described so far. In humans, there is only short-chain MK-4, which is a product of the systemic conversion of vitamin K_1_, and four forms of MK-7 to MK-10, products of the synthesis of bacteria [105,106,107,108,109,110,111,112]. The chemical structures of the known forms of vitamin K are shown in Figure 3.

The main sources of vitamin K_1_ are green plants and vegetable oils [106,110,113,114]. Meat, cheese, and fermented soybean products contain a form of vitamin K_2_ [106,108,110,114]. Since vitamin K is lipophilic, after incorporation into chylomicrons together with bile acids in this ω form, it enters the liver. Vitamin K metabolism in hepatocytes begins with ω-hydroxylation by cytochrome P450 4F2, followed by shortening of the polyisoprene chain and then β-oxidation to carboxylic acids at the C5, C7, or C10 position of the polyisoprene side chain. The resulting metabolites undergo glucuronidation in the mitochondria and are then removed from the body in the form of urine and bile [110,115,116]. The function of vitamin K is the post-ribosomal transformation of proteins dependent on this vitamin. In this protein transformation, vitamin K acts as a cofactor, and the carboxylated glutamic acid residues of the polypeptide chains of proteins produced are called Gla domains. Proteins with this domain are known as Gla proteins. The proteins activated in this process include coagulation factors II, VII, IX, and X [107,114]. Vitamin K-dependent proteins also include protein C, whose task is to prevent blood coagulation [107,117,118]; protein S, which serves as a cofactor for activated protein C and participates in the inactivation of factors Va and VIIIa of the coagulation process [107,118,119]; and protein Z, which also belongs to the factors of the blood coagulation cascade and is involved in the degradation of factor Xa [107,118,120]. Gla proteins are found throughout the body and are a component of bones as osteocalcin, matrix Gla protein (MGP) of the skeleton, kidney Gla protein, growth arrest-specific 6 protein which is involved in the stimulation of cell proliferation, and Gla-rich protein involved in the mineralization of soft tissues [106,108,121,122]. Vitamin K is involved in the process of preventing osteoporosis. Interleukin 6 (IL-6), which is an indicator of inflammation, also affects bone resorption in the process of osteoclastogenesis [123,124]. Vitamin K has been observed to decrease IL-6 production in human fibroblast cultures [125]. One of the important roles of vitamin K is the impact on osteoblast functions, i.e., proliferation, differentiation, and inhibition of apoptosis. The incorporation of organic and mineral matter into the bone matrix is also dependent on the level of alkaline phosphatase. Studies have shown that vitamin K increases the activity of alkaline phosphatase. In terms of osteoprotection, vitamin K participates in the activation of the steroid and xenobiotics receptor (SRX) and acts as a regulator of the transcription of osteoblast marker genes and extracellular matrix genes [109,126]. Vitamin K deficiency increases the risk of chronic inflammations [112]. Ferroptosis has been the focus of attention in the search for methods of cancer treatment. There is a link between ferroptosis and vitamin K. One of the effects of ferroptosis, as described in Section 3, is the formation of lipid peroxidation products. Vitamin K can be found in three forms in the body. These are the reduced (hydroquinone) form, the oxidized (quinone) form, and the epoxide form of vitamin K, respectively [109]. The reduced form of vitamin K inhibits lipid peroxidation by retaining free radicals in the plasma membrane. Vitamin K uses the activity of ferroptosis suppressor protein 1 (FSP1), with reductase activity at the expense of NAD(P)H, and thanks to it maintains a high level of the reduced form. This makes vitamin K have defined antioxidant properties [127,128]. In a significant study, Mishima et al. [127] have just shown that FSP1 maintains high levels of reduced vitamin K, thereby suppressing ferroptosis. In their studies on cell lines, the researchers indicate that that FSP1 is a crucial regulator of ferroptosis that operates separately from GPX4. This NAD(P)H-dependent ubiquinone oxidoreductase plays a significant role in ferroptosis by converting ubiquinone to ubiquinol and utilizing NAD(P)H [127]. Similar conclusions were reached by Kolbrink et al. [129], who showed that vitamin K_1_ is a strong ferroptosis inhibitor and can be used as a medicine in the course of acute kidney damage. Vitamin K_2_ has also been shown to inhibit ferroptosis and protect against cell death caused by oxidative stress. This effect appears to be mediated at least in part by activation of the Nrf2 antioxidant pathway. The Nrf2 pathway is a cellular defense mechanism that helps protect cells from oxidative stress by upregulating the expression of genes involved in antioxidant defense and detoxification. Vitamin K_2_ has been shown to activate this pathway, leading to increased expression of antioxidant enzymes, including glutathione peroxidase [130,131,132]. Given the potential role of ferroptosis in COVID-19 pathogenesis and the possible link between vitamin K and ferroptosis, there is interest in exploring the potential therapeutic benefits of vitamin K in COVID-19. However, more research is needed to fully understand the mechanisms underlying the relationship between vitamin K, ferroptosis, and COVID-19, and to determine whether vitamin K supplementation could be a safe and effective therapeutic strategy for COVID-19.

## 7. Vitamin K in COVID-19

The outbreak of the pandemic caused by SARS-CoV-2 prompted scientists to look for biologically active compounds that have antiviral and immunomodulatory effects. In patients with SARS-CoV-2, oxidative stress is associated with amplification and maintenance of the cytokine storm and coagulopathy [3]. COVID-19 induces a hypercoagulatory state that frequently leads to thromboembolic complications [133,134]. In hospitalized patients, accelerated elastic fiber from mineralization and degradation has been observed [135]. That is why vitamin K, which affects activation of anti- and proclotting factors in the peripheral tissues and liver, respectively, was of interest [136,137,138]. Vitamin K participates in the protection of the lungs against calcification and damage [137,139,140]. What is used for the identification of extrahepatic vitamin K status is dephosphorylated uncarboxylated matrix Gla protein (dp-ucMGP) and the ratio between uncarboxylated and carboxylated osteocalcin [135,137,141,142]. The high levels of dp-ucMGP reflect a low vitamin K status and vice versa [142]. Vitamin K deficiencies have been demonstrated in adults hospitalized with COVID-19 infection [135,138,141,143]. Low vitamin K status predicts higher mortality among patients with COVID-19 [138]. Because vitamin K has anti-inflammatory activity and offers protection against oxidative stress, it influences the course of the early phase of acute COVID-19 infection [141,143,144]. MGP, activated by vitamin K, inhibits the degradation of elastic fibers and vascular mineralization [135]. Increased use of vitamin K for carboxylation of pulmonary MGP and coagulation factors affect the course of the disease [135,138]. It is not low baseline vitamin K levels that are responsible for extrahepatic vitamin K deficiency, but mainly increased vitamin K use during infection [142].

The effects of vitamin K derivatives on human immune cells have not been extensively investigated. In an animal model, it has been shown that menadione may be an effective therapeutic strategy against acute lung injury, including ARDS. Vitamin K_3_ inhibits NF-κB activation, which is required for expression of cytokines and the following inflammatory responses in the ARDS mouse model (the mouse macrophage-like cell line RAW264.7) [145]. Vitamin K derivatives levels are inversely correlated with levels of inflammatory cytokines, including IL-6, tumor necrosis factor α (TNF-α) and C-reactive protein (CRP) [124,146]. They can increase the frequency of CD4^+^CD25^+^Foxp3^+^ regulatory T (Treg) cells, which play a vital role in maintaining immune homeostasis and in the prevention of autoimmune responses [147].

Although vitamin K deficiency is consistently associated with poorer clinical outcomes in COVID-19 patients, it has not been conclusively demonstrated that vitamin K supplementation has the potential to prevent or improve outcomes by increasing the activation of lung MGPs and endothelial protein S [135]. The investigated genetically predicted circulating vitamin K levels provide no evidence that vitamin K supplementation can prevent SARS-CoV-2 infection or hospitalization due to COVID-19 [136]. Regular use of vitamin K antagonists (VKAs) as anticoagulation drugs dramatically decreases the bioavailability of active vitamin K. Use of VKAs prior to COVID-19 is associated with increased mortality risk in COVID-19 patients [148]. Vitamin K deficiency is associated with factors that increase the risk of severe and fatal COVID-19, including demographics, body mass index (BMI), inflammatory markers, and comorbidities (cardiovascular, pulmonary, and renal diseases) [138,139,141,142]. Protective roles for vitamin K in inflammation may reduce the effect of adverse immune sequelae in the case of SARS-CoV-2 infection. Further research is required on the importance of vitamin K supplementation in the prevention and treatment of severe COVID-19.

## 8. Conclusions

COVID-19 is still the subject of numerous medical studies. Increased generation of ROS and RNS in the course of infection is associated with an unfavorable prognosis and a severe course of COVID-19 [149,150]. Imbalance in redox homeostasis leads to LPO, which, combined with disturbed iron metabolism in SARS-CoV-2 infection, promotes cell death by ferroptosis [84]. The use of vitamin K as a biomolecule participating in the coagulation and neutralization of ROS seems to bring therapeutic benefits and reduce ferroptosis. Fat-soluble vitamin K may limit the peroxidation of lipids that build cell membranes. A low level of vitamin K in the course of COVID-19 is associated with higher mortality [138].

Further medical research involving a larger group of COVID-19 patients is necessary to understand the mechanisms of ferroptosis and its role in disease progression and recovery. Among the many hypotheses and directions of research, the reduction of oxidative stress associated with COVID-19 seems a particularly promising strategy. The newly discovered antioxidant properties of vitamin K may prove particularly important in the treatment of SARS-CoV-2 infection. The investigation of the immunomodulatory role of vitamin K in COVID-19 patients is also a valuable research topic. Additional investigation is necessary to establish a direct association between the status of vitamin K and ferroptosis. This review publication may be an inspiration for taking up new, promising research directions regarding COVID-19 and oxidative stress. A better understanding of the mechanism of SARS-CoV-2 infection and the course of the disease will allow for the development of effective and safe therapies.
